# Current situation in Europe – different perspectives from Poland

**DOI:** 10.1192/j.eurpsy.2025.196

**Published:** 2025-08-26

**Authors:** J. Samochowiec

**Affiliations:** Department of Psychiatry, Pomeranian Medical University, Szczecin, Poland

## Abstract

**Abstract:**

The presence of large numbers of Ukrainians looking for refuge in Poland is a new experience for Poles. The ongoing war and the uncertainty of the situation of those displaced may cause anxiety and lead to stressful reactions, exacerbated by endlessly circulating information on hostilities. Therefore, the sense of security may be threatened not only among Ukrainians who have fled to Poland, but also among people who support Ukrainians, who offer them help and shelter. Prolonged support, if not accompanied by proper selfcare can increase the risk of burnout as well as lead to distressful emotional states, such as a feeling of helplessness, reluctance to provide further help, or even demonstrate hostility. The Polish government and polish NGO’s have pledged to help refugees from Ukraine, including the provision of mental health care. Raising awareness of the whole society and training employees from sectors other than medical may help in the proper protection of mental health of refugees and the people supporting them. Dividing the organization of mental health care into the four levels (Intervention Pyramid (Inter Agency Standing Committee, 2007)and offering support depending on the needs, ranging from building a basic sense of security, acceptance, and support for meeting the needs of refugees, to the level of highly specialized psychological and psychiatric assistance, enables the use of the resources of the entire society and specialists in an appropriate manner. By activating refugees themselves and training employees and volunteers of various sectors and fields of support, the goal of mental health promotion is spread across many environments, which mental health professionals themselves cannot cope with in these new, difficult conditions.

**Disclosure of Interest:**

None Declared

